# LINC00665 enhances tumorigenicity of endometrial carcinoma by interacting with high mobility group AT-hook 1

**DOI:** 10.1186/s12935-020-01657-2

**Published:** 2021-01-06

**Authors:** Yixuan Cai, Min Hao, Yue Chang, Yun Liu

**Affiliations:** Department of Obstetrics and Gynecology, Beijing Friendship Hospital Affiliated to Capital Medical University, Beijing, China

**Keywords:** Endometrial carcinoma, LINC00665, HMGA1

## Abstract

**Background:**

Endometrial carcinoma is a frequently diagnosed cancer among females. LncRNAs are reported to be associated with various cancers. Their biological roles in endometrial carcinoma progression is an emerging scientific area. LINC00665 can exert a significant role in many cancers. However, its potential function in endometrial carcinoma is still poorly known.

**Method:**

qRT-PCR was carried out to test expression of LINC00665 and HMGA1. Western blot analysis was carried out to detect protein expression of HMGA1. Cell proliferation was evaluated using Cell Counting Kit-8 (CCK-8) and EdU assay. Flow cytometry assay was used to determine cell apoptosis and cell cycle. Wound healing and transwell invasion assay was carried out to test cell migration and invasion. Immunohistochemical staining and HE staining were conducted to assess Ki-67 and tumor growth respectively.

**Results:**

Expression of LINC00665 in clinical endometrial carcinoma tissues and cells was obviously up-regulated. Loss of LINC00665 could repress endometrial carcinoma cell viability, induce cell apoptosis and block cell cycle in G1 phase. KLE and HHUA cell migration and invasion ability were depressed by LINC00665 shRNA. Decrease of LINC00665 suppressed endometrial carcinoma tumorigenicity in vivo. RIP assay proved that LINC00665 directly bound with HMGA1 protein. shRNA of HMGA1 obviously restrained endometrial carcinoma cell growth and cell invasion.

**Conclusions:**

LINC00665 might promote endometrial carcinoma progression by positively modulating HMGA1.

## Background

Endometrial carcinoma is a malignant tumor among women with a leading cause of mortality worldwide [1]. Endometrioid endometrial carcinoma is a frequent subtype of endometrial cancer, which can account for about 90% endometrial carcinoma cases [2]. The primary surgery in endometrial cancer is still generally not satisfactory [3]. Nevertheless, these treatments are not effective for advanced stage or distant metastases [4]. The molecular mechanism underlying endometrial cancer development remains poorly known. Therefore, it is of great significance to investigate the mechanism and identify effective therapeutic strategy for endometrial cancer.

LncRNAs are ncRNA transcripts with more than 200 nts crucial modulators in various cellular processes, including cell proliferation, cell apoptosis and cell cycle progression [5, 6]. Many lncRNAs are reported to be dys-regulated in human cancers [7–9]. Recently, the function of lncRNAs are widely described in endometrial carcinoma [10–12]. LncRNAs have been reported to act as diagnostic biomarkers used against endometrial cancer. For example, lncRNA TDRG1 can enhance endometrial carcinoma tumorigenicity via binding to VEGF-A protein [13]. LncRNA H19 can modulate the expression of HOXA10 in endometrial carcinoma by sponging miR-612 [14]. LncRNA GAS5 can increase PTEN expression via repressing miR-103 in endometrial cancer [15]. However, the molecular mechanisms underlying the role of lncRNAs in endometrial cancer require further investigation.

LINC00665 is located at chromosome 19q13.12 and many studies have demonstrated that LINC00665 serves as an oncogene in cancer progression. For example, microarray analysis has indicated LINC00665 is up-regulated in lung adenocarcinoma [16]. LINC00665 can promote prostate cancer development via miR-1224-5p and SND1 [17]. In addition, LINC00665 can induce the progression of multiple myeloma through modulating miR-214-3p and PSMD10 [18]. As stated above, the expression of LINC00665 is increased various cancers. However, the underlying mechanisms of LINC00665 in endometrial cancer needs more investigation.

In our current work, LINC00665 (ENST00000590622, NR_038278) was investigated. It was markedly elevated in endometrial cancer. The functions of LINC00665 in endometrial cancer have not been reported previously. Hence, we studied the impact of LINC00665 on the progression of endometrial cancer. Furthermore, mechanistic analysis indicated LINC00665 regulates the expression of HMGA1, thereby inducing endometrial cancer progression. These motivated us to explore the interaction between LINC00665 and HMGA1 in endometrial cancer development.

## Materials and methods

### Clinical samples

Ten endometrial carcinoma and 10 normal endometrial specimens were obtained from patients with surgical resection. No patients received any chemotherapy or radiotherapy before the surgery. Tissue samples were processed based on the ethical standards. We had obtained the informed consent form all the patients before enrollment. This research was approved by the Ethics Committee Beijing Friendship Hospital Affiliated to Capital Medical University.

### Cell culture

RL-95-2, Ishikawa, HEC-1B, KLE and HHUA cells and hESCs (human endometrial stromal cells) and were purchased from Cell Bank of the Chinese Academy of Science (Shanghai, China). DMEM added with 100 U/mL penicillin/streptomycin and 10% FBS. A 5% CO_2_ incubator at 37 °C was used to incubate the cells.

### Cell transfection

shRNAs for LINC00665 were constructed by Invitrogen (Carlsbad, CA, USA). 1 × 10^6^ cells per well were grown into six-well plates overnight and infected with the vector. The efficacy of transfection was tested using PCR. HMGA1 siRNA (RiboBio, Guangzhou, China). Lipofectamine 3000 (Invitrogen, CA, USA) was utilized to do cell transfection.

### CCK-8 assay

Cell viability was assessed by CCK-8 assay. 2000 cells were seeded into 96-well plates. Absorption was tested using a CCK-8 kit (Dojindo, Japan) at different time points. OD values were evaluated at 490 nm by a spectrophotometer (BioTek, Winooski, VT, USA).

### EdU assay

A Cell-Light EdU DNA Cell Proliferation Kit (KeyGEN BioTECH, China) was carried out to evaluate cell proliferation. Briefly, cells were grown into 96-well plates. Then, cells were treated with 50 µmol/L EdU for 2 hours. Then, cells were fixed using 4% paraformaldehyde and then 1 × Apollo reaction cocktail was used. Cell nuclei was stained using DAPI for 15 min. We captured the images using a fluorescence microscope (Nikon, Tokyo, Japan).

### Apoptosis assay

PI and FITC-labeled annexin V (BD Biosciences, New Jersey, USA) staining was used to assess cell apoptosis. Cells were washed twice using PBS and resuspended using 100 µL 1 × binding buffer, and then incubated with 5µL FITC-annexin V and PI with no light. Then, 400 µL 1 × binding buffer was used. Within 1 hour, cells were exposed to flow cytometry analysis.

### Cell cycle assay

Cells cultured in six-well plates, were trypsinized for cell cycle analysis. Then, after cells were washed using PBS, 70% ice-cold ethanol was added to cells at -20 °C for 2 hours for a whole night. Cell cycle detection kit and flow cytometry were used to determine cell cycle.

### Wound healing assay

Cells were seeded into 6-well plates. To form wounded gaps, cell layers were scratched using a 200 µL tip. Cells were cultured in FBS-free medium with 20 µg/mL mitomycin C. We photographed and analyzed the wounded gaps were at 0 and 24.

### Cell invasion assay

To carry out cell invasion experiment, we used matrigel Transwell Cell Culture chambers (BD Biosciences, San Jose, CA, USA). Matrix was put into the upper chamber of the chamber. 5 × 10^4^ cells were incubated in 200 µL FBS-free culture medium and then, added to the upper chambers. Then, 600 µL culture medium containing 10% FBS was placed to the lower chambers. Then, cells were fixed using 4% paraformaldehyde. Cells in the lower chamber were stained using crystal violet and imaged under an Olympus fluorescence microscope.

### qRT-PCR

TRIzol reagent (Takara, Tokyo, Japan) was used to extract total RNA. PrimeScript RT Reagent Kit (Takara, Tokyo, Japan) was employed to do reverse transcription. Then, SYBR green (Takara, Tokyo, Japan) was conducted to do qRT-PCR on Bio-Rad CFX96 system to test the expression of LINC00665 and HMGA1 mRNA. 2^−ΔΔCt^ method was carried out to analyze gene expression with primers listed in Table 1.

**Table 1:** Primers used for real-time PCR

Genes	Forward (5′–3′)	Reverse (5′–3′)
GAPDH	CAAGGTCATCCATGACAACTTTG	GTCCACCACCCTGTTGCTGTAG
HMGA1	GAAGGTGAAGGTCGGAGTC	GAAGATGGTGATGGGATTTC
LINC00665	GGTGCAAAGTGGGAAGTGTG	CGGTGGACGGATGAGAAACG

### Western blot

Protein was separated on 10% SDS polyacrylamide gels. Afterwards, proteins were transferred onto PVDF membranes. The membranes were blocked using 5% skimmed milk. Then, primary antibodies against HMGA1 and GAPDH (1:1000, CST, Boston, MA, USA) were used for a whole night. Next, the membranes were washed using TBST and incubated with the secondary antibodies (1:2000, CST, Boston, MA, USA) for 2 hours. Finally, we visualized the protein bands using the enhanced chemiluminescence reagent.

## RIP assay

RIP assay was performed using Magna RIP Immunoprecipitation Kit (Millipore, Bedford, MA, USA). Briefly, KLE cells were lysed using RIP lysis buffer. 100µL cell extract was incubated with magnetic beads conjugated to human anti-HMGA1 or normal mouse IgG.

### Animal assay

Twelve 8-week-old female mice were bought from Shanghai Animal Laboratory Center. The mice were maintained in specific conditions with no pathogen. Then, mice were divided into 2 groups and injected with 5 × 10^6^ KLE cells infected with LV-shLINC00665 or LV-NC, respectively. We measured tumor volume every week. 7 weeks later, the animals were sacrificed for future histopathological determination. Approval of this animal study was obtained from the Animal Research Ethics Committee of Beijing Friendship Hospital Affiliated to Capital Medical University.

### Histology

Tumor tissues were fixed using 4% paraformaldehyde and embedded in paraffin. H&E staining (Beijing Solarbio, Beijing, China) was carried out based on the manufacturer’s protocols. To carry out immunohistochemistry analysis, Ki-67 were detected in xenograft tumor tissues. The sections were deparaffinized, hydrated, and then antigen retrieved. Primary antibody (Ki-67, 1:500, Abcam, UK) was utilized and then a solution of anti-rabbit IgG was used for 15 min. Then, a 3,3-diaminobenzidine color kit was used. We captured the pictures using a light microscope.

### Statistical analysis

Data were analyzed by SPSS 20.0 (Chicago, USA) and GraphPad Prism 7 (GraphPad, CA, USA). Statistical significance between various groups was analyzing using Student’s t tests or one-way ANOVA analysis. P < 0.05 indicated the statistical significance.

## Results

### **Expression of LINC00665 in endometrial tissues and cells**

**Fig. 1:**
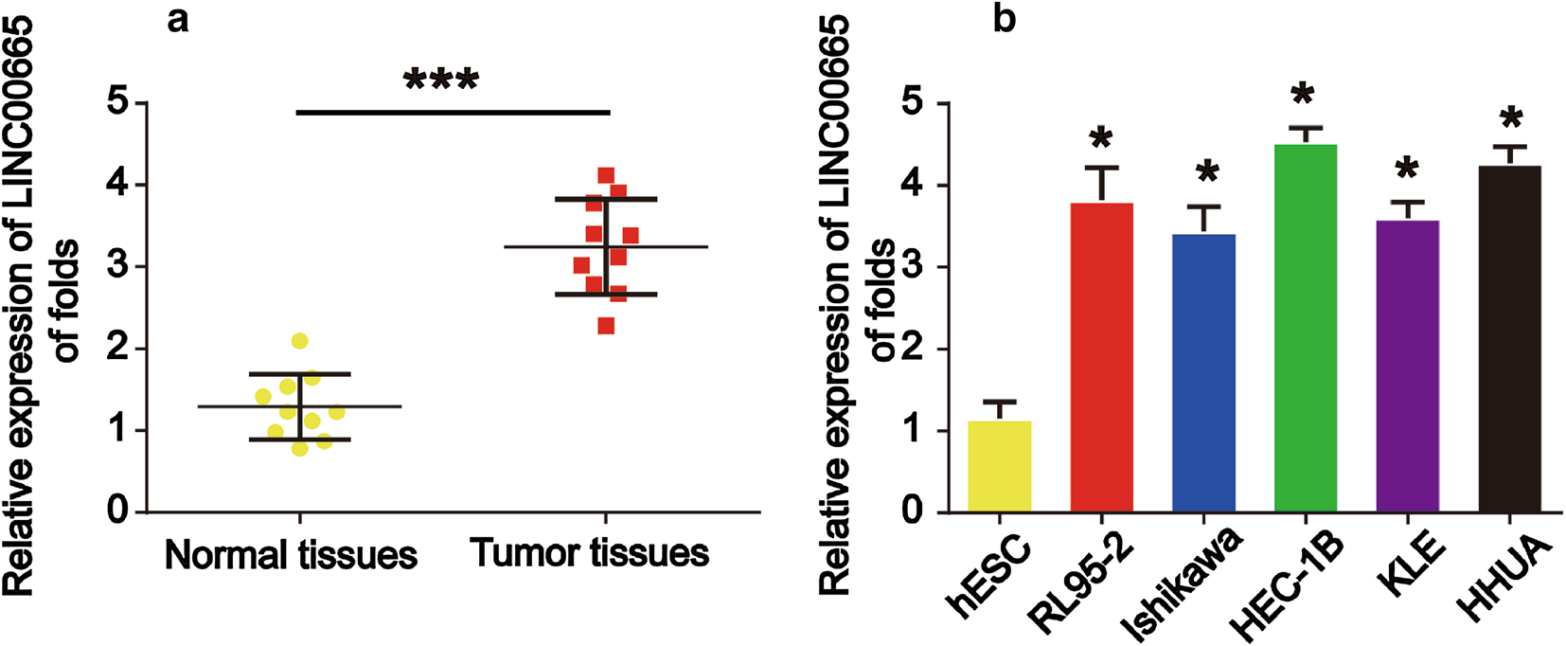
LINC00665 expression was up-regulated in endometrial cancer tissues and cell lines.** a** LINC00665 was highly expressed in endometrial cancer tissue than in normal ovarian tissues (ten pairs). **b** LINC00665 was highly expressed in endometrial cancer cellines (RL-95-2, Ishikawa, HEC-1B, KLE and HHUA cells) than in hESCs. Three independent experiments were carried out. Error bars stand for the mean ± SD of at least triplicate experiments. *P < 0.05, ***P < 0.001

First, to validate LINC00665 as a prognosis marker of endometrial carcinoma, we tested LINC00665 expression in endometrial carcinoma and normal tissues. Real-time PCR was used and we found that LINC00665 was obviously elevated in endometrial carcinoma in comparison to normal endometrial tissues as shown in Fig. 1a (P** < **0.001). In addition, we detected LINC00665 expression in commonly used endometrial cancer cell lines We found that LINC00665 was also increased in endometrial cancer cells (RL-95-2, Ishikawa, HEC-1B, KLE and HHUA cells) than in hESCs (Fig. 1b, P < 0.05). These indicated LINC00665 was significantly increased in endometrial carcinoma.

### LINC00665 repressed endometrial carcinoma cell proliferation

**Fig. 2:**
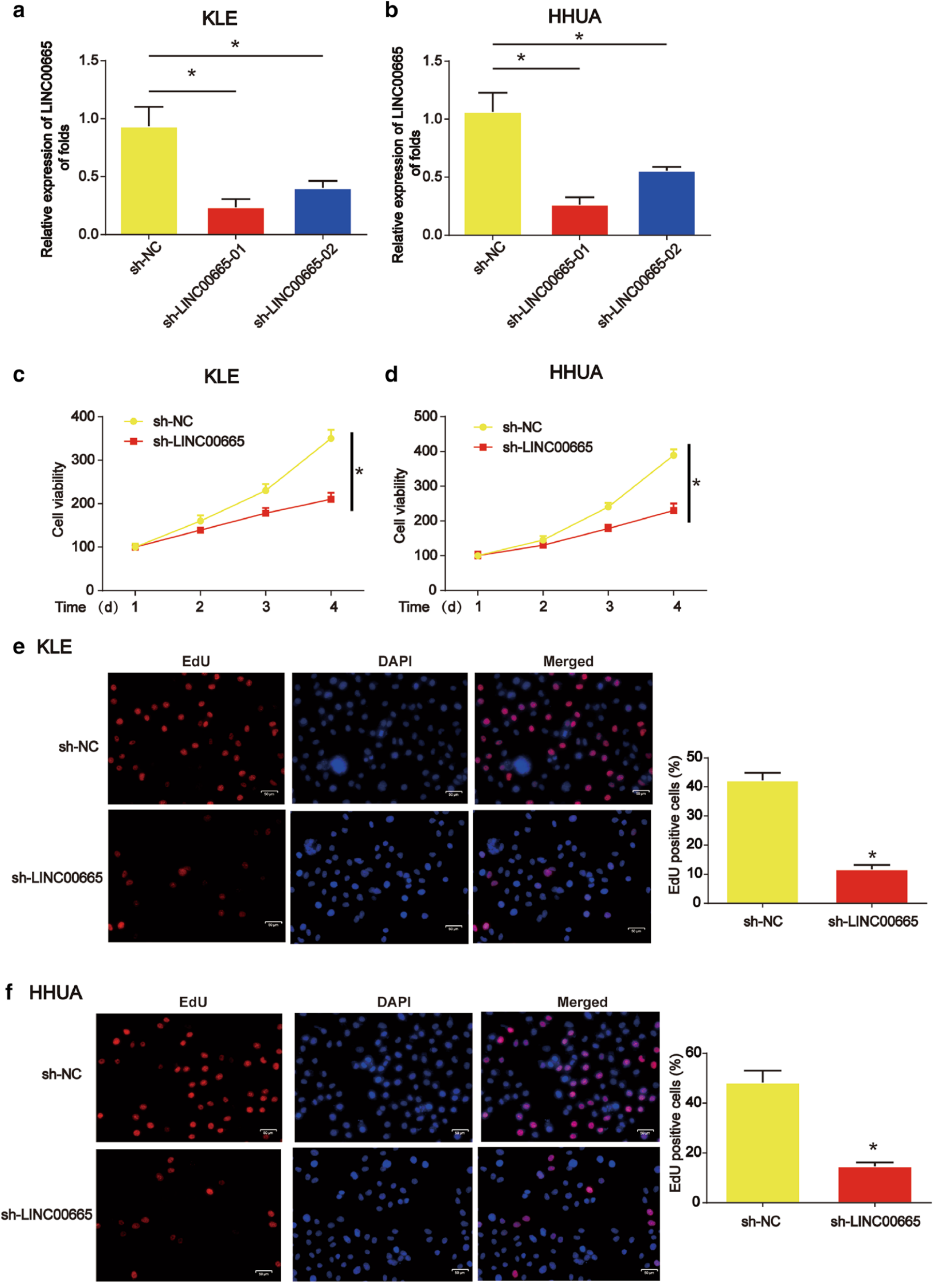
Loss of LINC00665 repressed proliferation and induced apoptosis in endometrial carcinoma cells. **a**, **b** LINC00665 expression in KLE and HHUA cells. Cells were infected with LV-shLINC00665 or LV-NC for 48 hours. **c**, **d** Effects of LINC00665 on KLE and HHUA cells survival. CCK-8 assay was conducted to detect cell viability. **e, f** Effects of LINC00665 on KLE and HHUA cell proliferation. EdU assay was performed to test cell proliferation. Three independent experiments were carried out. Error bars stand for the mean ± SD of at least triplicate experiments. *P < 0.05

Moreover, given that LINC00665 up-regulation in endometrial carcinoma, we next examined whether loss of LINC00665 inhibited endometrial carcinoma cell proliferation. Figure 2a and b revealed that LINC00665 was successfully down-regulated in KLE and HHUA cells after transfection with shRNAs (P < 0.05). In Fig. 2c and d, we found that decrease of LINC00665 significantly decreased KLE and HHUA cell survival as proved using CCK-8 assay (P < 0.05). Additionally, it was proved that KLE and HHUA cell proliferation was inhibited by the down-regulation of LINC0066 as quantified in Fig. 2e and f (P < 0.05).

### Knockdown of LINC00665 triggered endometrial carcinoma cell apoptosis and blocked cell cycle progression

**Fig. 3:**
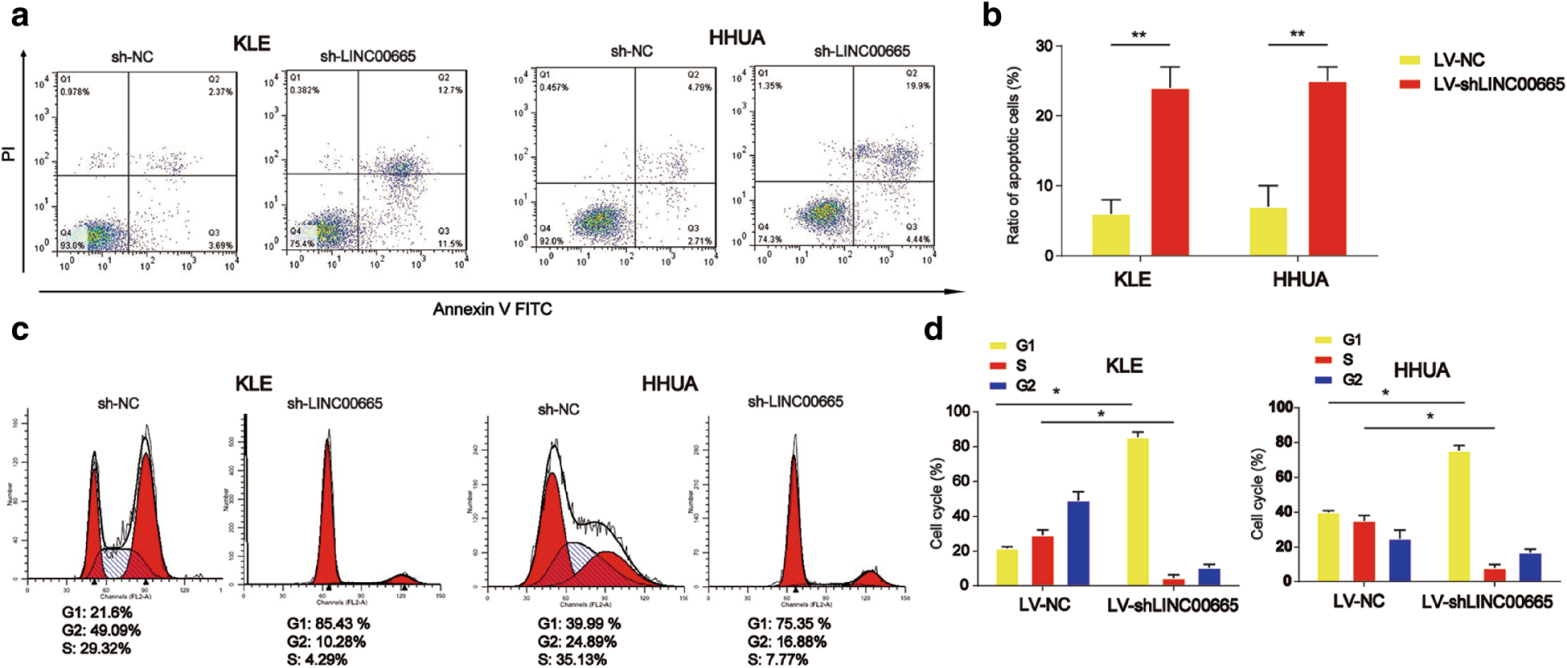
Effects of LINC00665 on endometrial carcinoma cell apoptosis and cell cycle. **a**, **b** Effects of LINC00665 on KLE and HHUA cell apoptosis. Flow cytometry was used to detect cell apoptosis. **c**, **d** Effects of LINC00665 on KLE and HHUA cell cycle. Flow cytometry was conducted to detect cell cycle. Three independent experiments were carried out. Error bars stand for the mean ± SD of at least triplicate experiments. *P < 0.05, ** P < 0.01

Next, in Fig. 3a and b, we demonstrated that KLE and HHUA cell apoptosis was induced by lack of LINC00665 as determined using flow cytometry analysis (P < 0.01). In addition, it was shown that LV-shLINC00665 greatly blocked KLE and HHUA cell cycle progression at G1 phase as exhibited in Fig. 3c and d (P < 0.05).

### Down-regulation of LINC00665 restrained endometrial carcinoma cell migration and invasion capacity

**Fig. 4:**
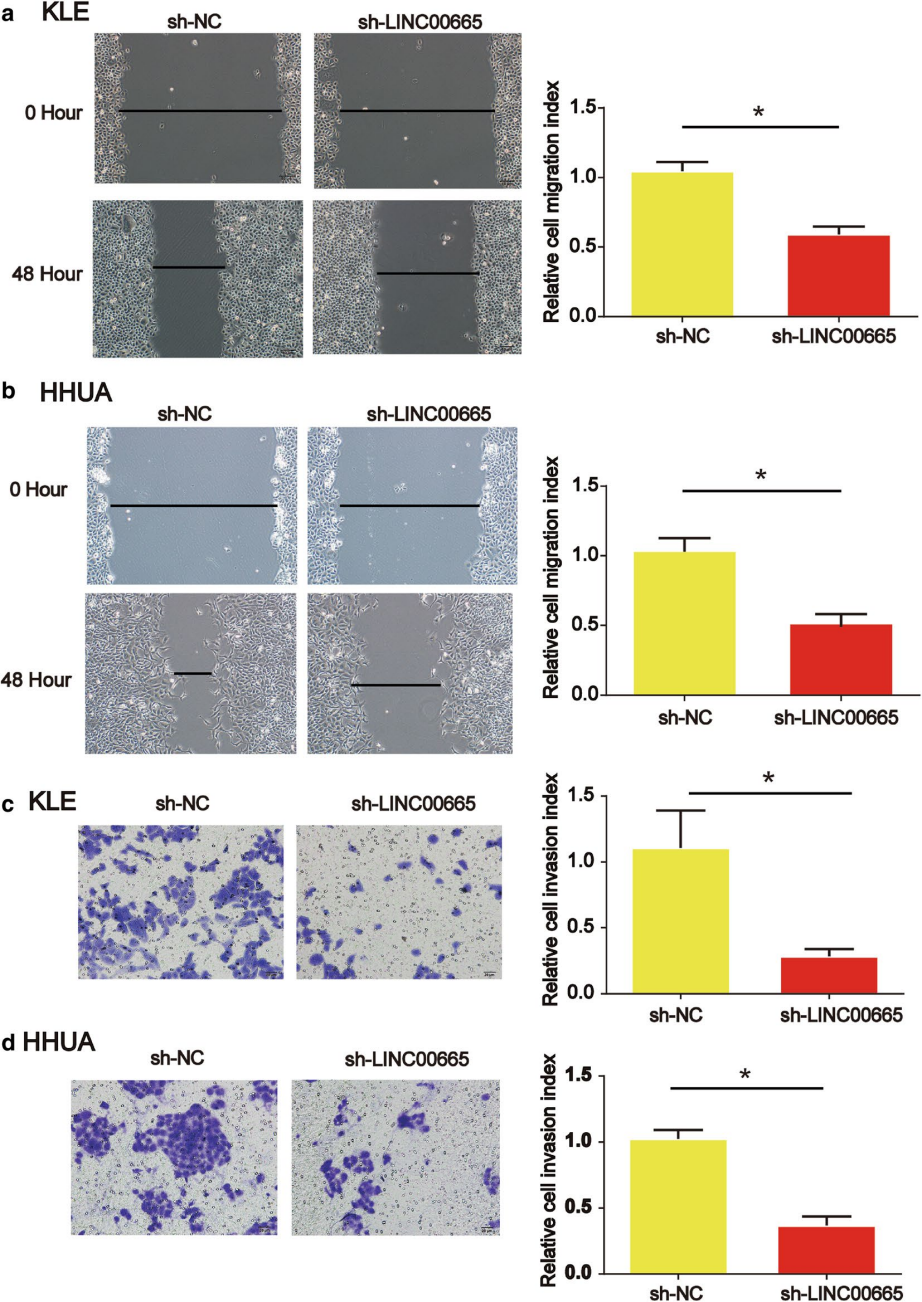
Effects of LINC00665 on endometrial carcinoma cell migration and invasion.** a**, **b** Effects of LINC00665 on KLE and HHUA cell migration. Would healing assay was performed to detect cell migration capacity. **c**, **d** Effects of LINC00665 on KLE and HHUA cell invasion. Three independent experiments were carried out. Error bars stand for the mean ± SD of at least triplicate experiments. *P < 0.05

Moreover, we evaluated the migration and invasion capability of KLE and HHUA cells by wound-healing assays and transwell invasion assay. In Fig. 4a and b (P  < 0.05), shRNA of LINC00665 significantly reduced the wound closure in KLE and HHUA cells. Then, transwell invasion assay indicated that decrease of LINC00665 greatly retarded KLE and HHUA cell invasion ability in Fig. 4c and d (*P* < 0.05).

### Silence of LINC00665 inhibited endometrial carcinoma cell growth in vivo

**Fig. 5:**
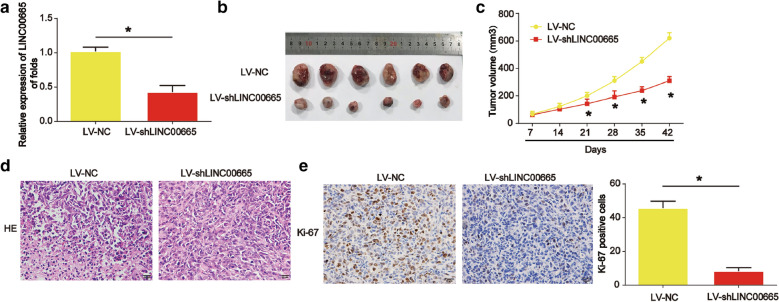
Loss of LINC00665 repressed endometrial carcinoma in vivo. Twelve 8-week old female BALB/c nude mice were injected with KLE cells infected with LV-NC (six mice) or LV-shLINC00665 (six mice). **a** Expression of LINC00665 in the tumor tissues. **b** Tumors isolated from the two groups. **c** Tumor volume. **d**, **e** IHC staining of Ki-67 in tumor tissues. Three independent experiments were carried out. Error bars stand for the mean ± SD of at least triplicate experiments. *P < 0.05

To explore the effect of LINC00665 on the endometrial carcinoma cells tumorigenesis in vivo, KLE cells silenced with LINC00665 were transplanted into nude mice subcutaneously. As displayed in Fig. 5a, due to application of Sh-LINC0065, its expression was significantly reduced in the tumor graft (*P* < 0.05). shRNA of LINC00665 was able to reduce tumor growth and tumor volume in Fig. 5b and c (*P* < 0.05). Furthermore, IHC analysis of xenografted tumors displayed LINC00665 shRNA significantly reduced cell growth and cell proliferation rate (Ki-67) as shown in Fig. 5d and e (*P* < 0.05)**.** Therefore, the data manifested that lack of LINC00665 suppressed endometrial carcinoma cell tumorigenesis in vivo.

### LINC00665 co-immunoprecipitated with HMGA1 protein

**Fig. 6:**
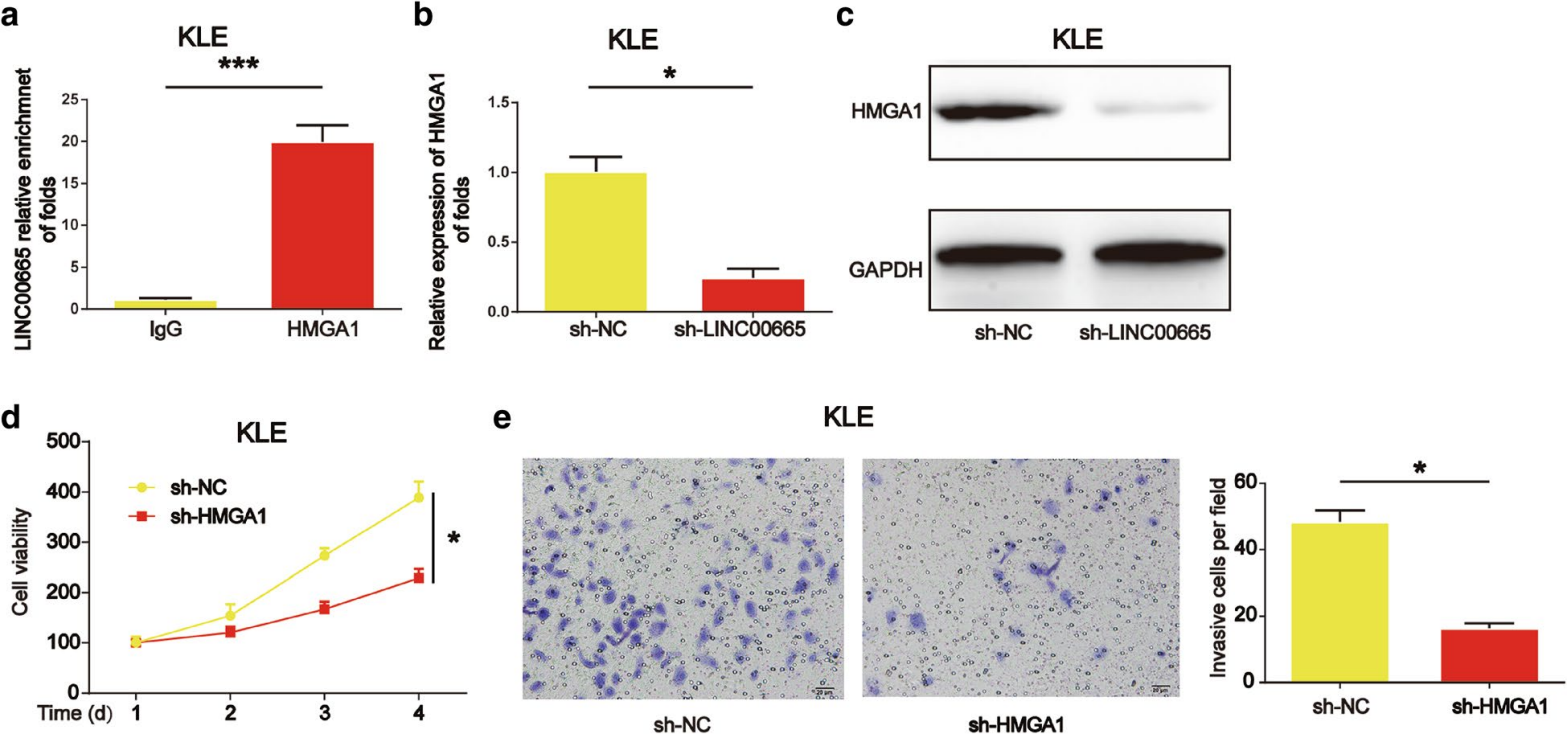
HGMA1 with LINC00665. **a** HMGA1 protein interacted with LINC00665. **b**, **c** HMGA1 mRNA and protein expression in KLE cells. **d** KLE cell proliferation. Cells were infected with HMGA1 siRNA. **e** KLE cell invasion. Three independent experiments were carried out. Error bars stand for the mean ± SD of at least triplicate experiments. *P < 0.05, ***P < 0.001

Subsequently, to explore the biological mechanism of LINC00665 in endometrial cancer, RIP assay was used to test whether HMGA1 protein interacted with LINC00665. RNA obtained from RIP assay using a HMGA1 antibody was exposed to qPCR analysis and an enrichment of LINC00665 was demonstrated (Fig. 6a, P < 0.001). Loss of LINC00665 significantly repressed HMGA1 mRNA and protein expression in Fig. 6b and c (*P* < 0.05). KLE cell viability and invasion was markedly reduced by HMGA1 siRNA in Fig. 6d and e (*P* < 0.05). Taken together, these data indicated a close interaction between LINC00665 and HMGA1 in endometrial cancer.

## Discussion

In recent years, lncRNAs are identified to be closely associated with many cancers [19, 20]. In addition, increasing studies have displayed ncRNAs are differentially expressed in endometrial cancer [21]. Numerous lncRNAs are altered in cancers and they have been linked to the progression of tumor cells. LncRNAs may represent candidate biomarkers for the diagnosis or treatment of cancers. LINC00665 can induce resistance to gefitinib via recruiting EZH2 and the activation of PI3K/AKT signaling in lung cancer [22]. LINC00665 can promote tumorigenesis of gastric cancer via regulating miR-149-3p and RNF2 [23]. In addition, LINC00665 contributes to breast cancer development via modulating miR-379-5p and LIN28B [24]. In hepatocellular carcinoma, LINC00665 modulates viability, apoptosis, and autophagy through sponging miR-186-5p and regulating MAP4K3 [25].

In our current research, we found LINC00665 was greatly up-regulated in endometrial cancer tissues and cells compared to the corresponding controls. In our future study, more endometrial cancer patients should be enrolled to observe their overall survival. Therefore, LINC00665 can serve as an important oncogene in the progression of endometrial cancer. Loss of LINC00665 repressed proliferation, G1-S progression, migration and invasion capacity and induced apoptosis in KLE and HHUA cells. Moreover, a tumorigenesis assay proved that silence of LINC00665 restrained endometrial tumor growth. This was consistent with the biological roles of LINC00665 in other cancers [22–24]. Hence, we explored the mechanism by which LINC00665 enhanced endometrial carcinoma tumorigenicity.

LncRNAs can be involved in various pathological processes, including various cancers. LncRNAs can interact with both nucleic acids and proteins directly [26]. LncRNAs exert significant roles in regulating protein expression [27–29]. In this study, we found that many miRNAs contained binding sites with both LINC00665 and HMGA1, which indicated that there might be an interaction between them. Through carrying out RIP assay, we observed that LINC00665 interacted with the HMGA1 protein. Additionally, silencing HMGA1 in KLE cells reduced the proliferation and invasion capacity. These results suggested LINC00665 might regulate endometrial carcinoma by interacting with HMGA1.

Studies have shown that the silence of HMGA1 represses cancer development [30, 31]. For example, HMGA1 can promote breast cancer angiogenesis via supporting the transcriptional activity of FOXM1 [32]. HMGA1 exacerbates cervical cancer growth via regulating cell cycle and migration/invasion capacity through targeting miR-221/222 [33]. HMGA1 is correlated with breast cancer malignant status [34]. In addition, HMGA1 can facilitate endometrial cancer progression by regulating Wnt/β-catenin signaling [35]. Thus, we suggested that LINC00665 may induce tumorigenesis by binding with HMGA1 in endometrial carcinoma. In our future study, we would like to perform an overexpression of HMGA1 experiment and then observe its effect on endometrial carcinoma cell growth to strengthen our data.

## Conclusions

This study demonstrated LINC00665 promoted endometrial carcinoma tumorigenesis and progression through interacting with HMGA1. These data could pave a new way to develop novel diagnostic and treatment strategy for endometrial carcinoma.

## Data Availability

Not applicable.
